# Functional Cognitive Disorder and Post-Concussion Memory Symptoms as Disorders of Distributed Memory Control: A Metacognitive–Systems Consolidation Framework

**DOI:** 10.3390/brainsci16101026

**Published:** 2026-09-25

**Authors:** Ioannis Mavroudis, Oindrila Das, Foivos Petridis, Dimitrios Kazis

**Affiliations:** 1 Department of Neuroscience, Leeds Teaching Hospitals NHS Trust, Leeds LS1 3EX, UK; 2 Faculty of Medicine, University of Leeds, Leeds LS2 9JT, UK; 3 Third Department of Neurology, Aristotle University of Thessaloniki, 54124 Thessaloniki, Greece; 4 Independent Researcher, Gurgaon 122018, India

**Keywords:** functional cognitive disorder, persistent post-concussion symptoms, metacognition, systems consolidation, anterior thalamus, predictive processing, overfitted brain hypothesis, maladaptive neuroplasticity, neurometabolic cascade, functional neurological disorder

## Abstract

**Background:** Memory problems are amongst the most troublesome and least understood of the symptoms in functional cognitive disorder (FCD) and in persistent post-concussion symptoms (PPCS). Standard neuropsychological testing typically shows only minor objective deficits, which results in a clear mismatch between the level of subjective distress and the actual degree of impairment. Current explanations of this discrepancy either attribute it to undetected hippocampal storage failure or regard it as a non-specific functional overlay; we believe that both of these explanations are incomplete. **Methods:** This is a narrative review incorporating an integrative hypothesis, not a systematic review. The literature was found using searches of databases and by tracing citations, and the sources were chosen on the basis of their conceptual relevance rather than according to a comprehensive protocolMethods of Literature Selection. The following three types of claims are distinguished throughout: those findings which have been established empirically in humans, those which are based on animal studies, and the hypotheses put forward in this paper. **Results:** Current evidence shows that episodic memory is spread out over a hippocampal–entorhinal–prefrontal–anteromedial thalamic–cortical control system, with the anteromedial thalamus being responsible for deciding which traces are stabilised and the prefrontal areas providing support for retrieval and for metacognitive evaluation. We suggest that the memory symptoms in both cases are due to a malfunction of this control system rather than to a failure of storage. In the case of FCD, entry is thought to occur in a top-down manner via abnormally precise priors of cognitive failure, impaired global metacognition, hypervigilant self-monitoring and overfitting of the self-model. With regard to PPCS, entry is believed to take place in a bottom-up way as a result of a real neurometabolic and network injury, after which fatigue, sleep disruption and hypermonitoring keep the symptoms going; in susceptible individuals, a concussion may serve as a gateway to FCD. Maladaptive, use-dependent plasticity is proposed as the common underlying mechanism. **Conclusions:** A unified framework incorporating metacognitive and systems consolidation processes is able to explain the discrepancy and makes eight predictions that can be tested together with provisional criteria for the transition from a primary-FCD to an organic-PPCS. The framework is put forward for future testing rather than being presented as a fully established explanation.

## 1. Introduction

Subjective memory complaints are among the most common presenting symptoms in cognitive neurology clinics and are particularly prominent in two superficially distinct conditions: Functional Cognitive Disorder (FCD) and persistent Post-Concussion Symptoms (PPCS), which is defined here as symptoms persisting beyond the expected recovery window of three months [1,2,3,4]. Both groups of patients report disabling forgetfulness, word-finding difficulty, distractibility and a perception of profound mnemonic decline; both typically perform within or close to normal limits on standard neuropsychological testing; and both endure prolonged disability that is poorly explained by available objective findings [5,6,7,8].

The conceptual response to this gap has so far been bifurcated. Within neurology, FCD has been increasingly recognised as a disorder belonging to the functional neurological disorder (FND) spectrum, characterised by internal inconsistency between subjective complaint and objective performance, and framed as a disorder of metacognition, attention and predictive processing [1,2,3,9,10,11]. PPCS, by contrast, has remained anchored to the biomechanics of mild traumatic brain injury (mTBI), with persistence interpreted as either subtle but lingering network injury or, more recently, as a functional overlay layered on a recovering but vulnerable brain [6,12,13,14,15].

Common among these conditions is a marked discrepancy: the severity of the complaints about memory and the extent to which they are disruptive being consistently greater than the results of objective neuropsychological testing, which usually lies within normal ranges. We contend that this discrepancy is best explained not by invoking undetected hippocampal storage failure but by viewing both conditions as disorders of distributed memory control. We adopt as our scaffold the contemporary view of episodic memory as the product of a coordinated hippocampal–prefrontal–anterior thalamic–cortical network whose function depends critically on attention, retrieval scheduling, sleep-dependent consolidation, metacognitive evaluation and ongoing synaptic plasticity [16,17,18,19,20,21,22,23]. We further integrate this with predictive processing accounts of FND [9,11,24], the Overfitted Brain Hypothesis [25,26], and the concept of maladaptive neuroplasticity [27,28,29] to articulate a unified framework.

Section 2 summarises the distributed memory model. Section 3 applies it to FCD, including a new subsection developing the overfitting account in parallel with prior-update and attentional accounts. Section 4 addresses PPCS, beginning with an expanded treatment of the initial biological injury—the neurometabolic cascade and its impact on the very plasticity machinery that the memory system depends on—and arguing, following our recent work [30], that concussion may serve as a gateway to FCD in vulnerable individuals. Section 5 develops the cross-cutting role of maladaptive neuroplasticity. Section 6, Section 7, Section 8 and Section 9 develop the unified model, testable predictions, differential diagnosis, and clinical implications.

### Methods of Literature Selection

This article offers an integrative hypothesis in the form of a narrative review; it was not carried out as a systematic review since no protocol was registered, no formal assessment of the quality was performed, and no effort was made to carry out a comprehensive retrieval. The literature was identified through searches of PubMed/MEDLINE, Scopus and Google Scholar conducted in August 2026, covering publications from January 2000 to August 2026, supplemented by manual reference-list review and forward citation tracking. The search terms were combined across three different areas: the clinical syndromes (“functional cognitive disorder”, “functional memory disorder”, “post-concussion syndrome”, “persistent post-concussion symptoms”, “mild traumatic brain injury”, “functional neurological disorder”); the memory-control architecture (“systems consolidation”, “anterior thalamus”, “anteromedial thalamus”, “memory gating”, “episodic retrieval”, “metacognition”); and the mechanistic accounts cited (“predictive processing”, “overfitted brain hypothesis”, “maladaptive neuroplasticity”, “neurometabolic cascade”). The reference lists of the main articles were manually checked and forward citation tracking was carried out on the key sources. The sources were chosen according to their conceptual relevance to both the memory-control architecture and the two clinical syndromes; preference was given to primary studies involving humans, to systematic reviews where these were available, and to animal studies at the circuit level that had a direct mechanistic relevance; in the case where more than one source made the same point, the most recent one and the one with the strongest methodology were cited; publications that were not in English and conference abstracts were excluded. The fact that a large language model was used to help with literature retrieval and the organisation of references is set out in the declarations. The authors checked all the sources obtained in this way against the original publications and never cited any source on the basis of a summary produced by the model. Since the selection was conceptual and not exhaustive, this review is vulnerable to selection bias and the balance of the evidence cited should not be taken as representing the true balance of the evidence in the field; the framework is therefore to be evaluated in terms of the falsifiability of the predictions it makes, as outlined in Section 7, rather than in terms of the completeness of the literature reviewed.

## 2. The Distributed Memory Control System

Although the hippocampus retains a privileged role in the rapid encoding of episodic and contextual information [21,22], a substantial body of cellular and systems-level neuroscience now positions it as one node within a wider control architecture. Five features of this architecture are central to the present argument.

### 2.1. Rapid Encoding and the Entorhinal–Hippocampal Interface

Episodic memories are initially formed through rapid synaptic plasticity within hippocampal circuits, supported by patterned inputs from the entorhinal cortex that carry spatial, sensory and contextual information [21,31]. Crucially, however, prefrontal engram cells are generated essentially simultaneously with hippocampal ones during initial learning, even though they remain functionally silent for some time before maturing [17,22]. The classical view of a temporally extended transfer of memories from hippocampus to cortex has therefore been refined: cortical traces are present from the outset, and systems consolidation reflects their gradual maturation under continued input from medial temporal, thalamic and limbic structures [17,18,22,23].

### 2.2. The Anterior Thalamus as a Gate

***Evidence from animal studies:*** Toader and his team demonstrated, by means of simultaneous cell-resolution imaging in mice over a period of weeks of training, that the anteromedial thalamus has a sustained correlate of memory such that when this correlate is inhibited, consolidation is disrupted, whereas increasing its strength can save memories that would otherwise not consolidate [16]. Further research involving rodents also indicates that inhibitory projections from the hippocampus to the thalamus have a time-dependent function in the retrieval of remote memories [32]. These results suggest the existence of a thalamo-cortical circuit that actively selects the traces to be stored in the cortex in a durable manner.

***Evidence from human studies:*** Long-standing lesion studies have associated damage to the diencephalon with amnesic syndromes [19,20,33], and the anterior and mediodorsal nuclei are known to be closely interconnected with the salience network—that is, the dorsal anterior cingulate and anterior insular system which detects behaviourally significant events and determines access to controlled processing [34,35]. Accordingly, the anterior thalamus should best be regarded not as a relay but as a control structure, playing a role in the temporal organisation of memory networks and in integrating hippocampal output with that of the prefrontal and cingulate areas [19,20,36]. Nevertheless, there are no human studies showing a gating function at the circuit level.

***Hypotheses and predictions:*** Two of the extensions are purely hypothetical. In the first place, the animal evidence shows that gating occurs during consolidation; our framework stipulates that gating should also occur during retrieval, and this extension from consolidation to retrieval is taken for granted rather than being proven. Secondly, the idea that this gate is miscalibrated in FCD is put forward in this paper and has not yet been observed. To test this hypothesis, it would be necessary to carry out functional imaging studies in humans, looking at the engagement of the anteromedial thalamus during cued retrieval in FCD as compared with healthy and neurodegenerative control groups, with the prediction that the thalamic–prefrontal coupling during retrieval will be altered whereas hippocampal encoding activity will be unchanged. If that dissociation is not found, it would cast doubt on the framework.

### 2.3. Prefrontal Cortex, Metacognition and Retrieval

The prefrontal cortex contributes to memory in two interlocking ways. First, it organises cue-guided retrieval, holds task context online, and supports the selection of relevant traces against competitors [18,22]. Second, it provides the substrate for metacognition—the second-order evaluation of one’s own cognitive performance. Anterior prefrontal cortex, in particular, supports the formation of confidence judgements and the evaluation of mnemonic outputs, with damage in this region producing selective deficits in metacognitive accuracy without affecting first-order task performance [37,38,39]. A Bayesian framework formalises this role: metacognitive evaluation can be modelled as inference on the reliability of one’s own internal states, integrating prior expectations with evidence about ongoing performance [40,41]. Metacognition operates at multiple temporal scales: local metacognition refers to trial-by-trial confidence judgements; global metacognition refers to long-run beliefs about one’s own cognitive ability [1,42]. These are partially dissociable: a person can have intact local confidence calibration on a single task while holding markedly distorted long-run beliefs about their own memory [1,42].

### 2.4. Sleep-Dependent Consolidation and Generalisation

Sleep is the fourth essential component. During slow-wave sleep, slow oscillations, spindles and ripples coordinate the reactivation of recently encoded traces and their redistribution to neocortical circuits, while rapid eye movement (REM) sleep supports synaptic consolidation in cortex [43,44]. Disruption of these processes degrades the precision and accessibility of memories even when waking encoding is preserved [43,44]. A complementary recent proposal—the Overfitted Brain Hypothesis [25]—argues that dreaming serves a specific computational function: it injects out-of-distribution, hallucinatory samples that prevent neural networks from over-fitting to the narrow distribution of waking experience, thereby preserving the brain’s capacity to generalise. On this account, sleep is not only a consolidation phase but a regularisation phase. Disruption of sleep and dreaming therefore has two costs: degraded consolidation of recent traces, and degraded generalisation of internal models. We return to this in Section 3.5.

### 2.5. Synaptic Plasticity as Substrate

Underlying all of the above is synaptic plasticity. Long-term potentiation and depression at hippocampal, cortical and thalamo-cortical synapses, mediated by NMDA-receptor signalling, calcium-dependent kinases (CaMKII), CREB-dependent transcription and BDNF-mediated structural change, are the cellular currency of learning, consolidation and retrieval [27,45,46]. Sleep-dependent synaptic homeostasis renormalises this plasticity nightly, preserving signal-to-noise across the system [43]. Crucially, the same plasticity machinery that supports adaptive learning is the substrate by which dysfunctional patterns can become entrenched—a point we develop in Section 5.

### 2.6. Summary

Episodic memory function depends on at least five interacting components: an entorhinal–hippocampal encoder; a prefrontal–thalamic control hub that schedules and selects what is stabilised; a cortical long-term repository; a sleep-dependent consolidation and regularisation process; and a metacognitive evaluator that monitors and reports on the quality of mnemonic outputs. Synaptic plasticity is the cellular substrate that ties these components together. This architecture provides the template for understanding both FCD and PPCS.

## 3. Functional Cognitive Disorder as a Disorder of Distributed Memory Control

FCD is defined by persistent, distressing subjective cognitive symptoms—most often memory complaints—that are inconsistent with objective performance and not better explained by another neurological, psychiatric or systemic illness [1,2,3,6,8]. Internal inconsistency is the diagnostic linchpin: patients describe profound forgetfulness yet recall their own clinical history fluently; they fail simple bedside tasks while succeeding on harder versions; and their immediate recall may be poorer than delayed recall, a pattern atypical of neurodegeneration [2,6,47]. Recent reviews have systematised these features and proposed structured criteria [2,3,6,48,49].

### 3.1. The Metacognitive Disturbance

The most influential mechanistic account positions FCD as primarily a disorder of metacognition [1,50,51,52,53,54]. Bhome and colleagues, using hierarchical Bayesian modelling of confidence ratings, found that patients with FCD showed intact local metacognitive efficiency on perception and memory tasks but markedly impaired global metacognition—they significantly underestimated their cognitive ability on the Multifactorial Memory Questionnaire compared with normative data [1]. Pennington and colleagues reported convergent findings: trial-by-trial confidence calibration was largely preserved in FCD relative to neurodegenerative mild cognitive impairment [42]. Larner and others have argued that this dissociation between local and global metacognition is itself a candidate diagnostic feature of FCD [51,53].

The pattern is more in line with failed retrieval and monitoring than with failed storage, although this conclusion needs to be qualified. Since neuropsychological testing is only poorly able to distinguish among encoding, storage, retrieval, and metacognitive monitoring, factors such as reduced effort, attentional fluctuations, and low motivation can result in a similar pattern without necessarily involving a deficit in retrieval control. The studies in this area of research have generally not been designed to rule out these alternative explanations.

Our own work supports and extends these findings. In a study of reaction time, subjective mental effort and global metacognition, patients with FCD showed disproportionately high subjective effort and reduced global self-evaluation despite reaction-time profiles that remained close to normal [50]. In our recent synthesis [3], we argued that this pattern reflects a stable cluster of metacognitive distortions: catastrophic interpretations of normal cognitive lapses, perfectionistic memory standards, fear of underlying neurodegeneration, and avoidance of cognitively demanding situations.

A valid objection is that questionnaire-based scales assessing global self-evaluation might be measuring general distress or personality traits such as neuroticism and perfectionism rather than a specific metacognitive disturbance [55]. This worry is fully justified when looking at the global measures by themselves, but it is not so easily applied to the dissociation itself: local metacognitive efficiency is obtained from trial-by-trial confidence that is adjusted in relation to actual task performance rather than being based on self-description, and there is no reason why a theory based on distress or personality traits should leave this aspect unaffected while global self-evaluation is considerably impaired. Although the objection points out a real deficiency, since previous studies have not taken neuroticism, perfectionism or present mood into account, the local–global dissociation should therefore still be found after statistical adjustments have been made for these factors; if it is not, then the metacognitive explanation as given here must be rejected in favour of one that is mediated by distress.

### 3.2. Aberrant Priors and the Failure to Update

Within a predictive processing framework, the FCD metacognitive distortions can be understood as aberrantly precise prior beliefs about cognitive failure [9,11,24]. The brain enters each cognitive encounter with a strongly weighted prediction that performance will be poor; sensory evidence to the contrary—successful recall, intact recognition, fluent conversation—is down-weighted as noise, while every benign lapse is over-weighted as confirmatory evidence. The result is a system whose posterior beliefs barely update even in the face of disconfirming data [9,24,56]. This failure to update priors is not unique to FCD; it is now a leading mechanistic account across functional neurological symptoms more broadly [9,11,24].

### 3.3. Attention, Monitoring and Retrieval Inefficiency

FCD is not, however, only a disorder of belief. Teodoro and colleagues, in a unifying review of cognitive abnormalities across FND, fibromyalgia and chronic fatigue syndrome, found that subjective complaints far outstripped objective deficits, but that genuine impairments did exist—particularly in selective and divided attention, processing speed and vulnerability to distraction [57]. They proposed that excessive monitoring of internal cognitive processes itself consumes attentional resources and degrades performance, producing a self-reinforcing loop in which attempts to monitor memory worsen the very memory they are meant to track [57]. Translated into our framework, this maps onto prefrontal retrieval inefficiency rather than hippocampal storage failure: the traces exist, but the prefrontal scheduler is overloaded by self-monitoring.

### 3.4. Brain Fog and the Experience of Cognitive Failure

McWhirter and colleagues have argued that the lay term “brain fog” describes a coherent phenomenology—slowed thinking, mental effortfulness, and a sense of cognitive opacity—that overlaps substantially with FCD and is recognisable across chronic illnesses [58]. This phenomenology is precisely what a predictive processing model would predict from a metacognitive controller that has lost calibration: subjective effort outstrips objective demand, and the experience of mnemonic difficulty becomes detached from actual mnemonic capacity [3,9,11].

### 3.5. FCD as an Overfitting Disorder

The accounts above—metacognitive distortion, aberrantly precise priors, attentional hijacking and maladaptive behaviour—are convergent rather than competing. We propose that they can be integrated, and substantially extended, by reading FCD as an overfitting disorder.

Hoel’s Overfitted Brain Hypothesis [25] reframes dreaming as the brain’s solution to a problem familiar from deep learning: any network trained on a narrow, highly correlated distribution of inputs will fit that distribution too closely and lose the ability to generalise. In artificial networks this is mitigated by injecting noise—dropout, randomised samples, out-of-distribution data—during training. Hoel argues that dreams, with their sparse, hallucinatory and combinatorial character, perform exactly this regularising function for biological brains [25,26]. Disruption of dreaming therefore degrades generalisation, even when memorisation is preserved.

Larner extended this hypothesis to FCD [59]. He observed that sleep disturbance is a near-universal feature of FCD, that subjective complaints in FCD typically reflect failures of flexible, generalised use of memory rather than focal recall deficits, and that the mismatch between memory expectations and memory performance is precisely what would be predicted if the underlying network had overfit to a narrow training distribution dominated by illness, threat and failure [59]. On this reading, the FCD brain has “learned” too well—but the lesson it has learned is wrong.

It must be stressed that this account is an extension of a theoretical suggestion and not the result of an empirically proven mechanism. Although sleep disturbance is well known to be associated with FCD, no study has shown that it plays a causal role in causing or sustaining the functional cognitive symptoms, and the specific assertion that dream-mediated regularisation is impaired in FCD has not been tested directly.

Read clinically, overfitting predicts a specific and observable signature: behavioural rigidity, or the failure to generalise demonstrably intact capacity across contexts. In machine learning, an overfitted model is precisely one that cannot generalise beyond its training distribution; in FCD, this manifests as the internal inconsistency that defines the disorder—a patient who fails a structured bedside memory task yet fluently recounts a detailed, temporally ordered history moments later [2]. The same self-report/performance dissociation is quantifiable in functional motor disorder, where patients estimated tremor was present for 83.5% of the waking day while actigraphy recorded 3.9%, the internal model rather than the sensory data governing the report [60]. Excessive self-focused monitoring, which forces a shift from automatic to effortful processing, supplies the mechanism by which fluent, generalised use of memory is lost [57].

This overfitting account does substantial work within our framework. First, it gives a mechanistic interpretation of aberrant priors: a network that has overfit to a self-model centred on cognitive failure will assign extremely high precision to predictions consistent with that model, while discounting contradictory evidence as out-of-distribution noise—precisely the pattern that predictive processing accounts of FND describe phenomenologically [9,24,56,59]. Second, it links the metacognitive and sleep elements of FCD: impaired sleep degrades both consolidation (Section 2.4) and the regularisation function of dreaming (Section 2.4; [25,26,59]), allowing overfitted internal models to persist uncorrected. Third, it provides a clinical target: interventions that broaden the distribution of cognitive evidence the system encounters—graded successful retrieval, behavioural experiments that violate the predicted-failure prior, and protected sleep and dreaming—are predicted to reduce overfitting rather than simply argue with the patient’s beliefs. This also explains a phenomenon that frequently frustrates clinicians: the failure of standard reassurance and normal test results to relieve symptoms. If the illness prior is granted excessive precision, disconfirming evidence—a normal scan, a passed cognitive test—is down-weighted as out-of-distribution noise rather than assimilated. Perceptual-dysregulation accounts describe exactly this attenuation [9,24]. The framework therefore predicts that reassurance delivered without first reducing the precision of the prior will be therapeutically inert, whereas interventions that re-weight the prior should restore responsiveness to normal findings.

The four mechanistic threads in FCD—impaired global metacognition, failure to update priors, attentional self-monitoring, and maladaptive avoidance behaviour—can therefore be read as different surfaces of a single underlying problem: an overfitted metacognitive controller embedded in a sleep-degraded distributed memory system, in which the prefrontal evaluator has learned a narrow, self-confirming model of its own failure.

**Figure 1:**
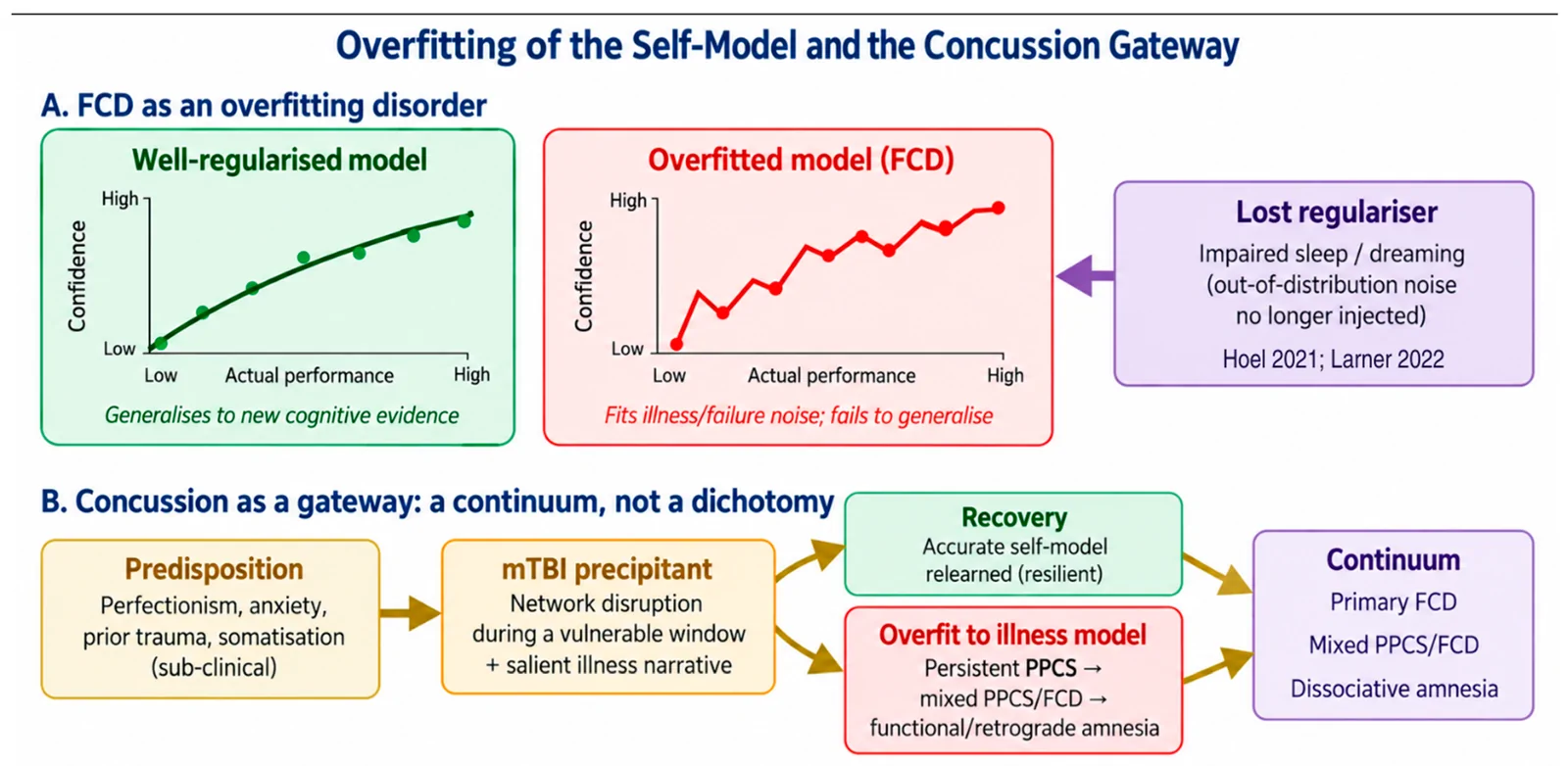
Proposed overfitting account of functional cognitive disorder. (**A**) In each panel, subjective confidence is plotted against actual cognitive performance. A well-regularised internal model generalises to new cognitive evidence, whereas an overfitted model fits illness- and failure-related noise and fails to generalise; impaired sleep and dreaming are hypothesised to remove the out-of-distribution regulariser (Hoel [25]; Larner [59]). (**B**) In vulnerable individuals, sub-clinical predisposition together with a mild traumatic brain injury precipitant acting during a vulnerable window, alongside a salient illness narrative, is proposed to drive overfitting rather than accurate relearning, along a continuum from primary FCD, through mixed PPCS/FCD, to dissociative-amnesia presentations. Both panels depict hypothesised relationships, not established findings.

Figure 1 summarises this account, together with the continuum it predicts when concussion acts as a precipitant.

### 3.6. FCD and Subjective Cognitive Decline

Cappa has recently argued that the boundary between FCD and subjective cognitive decline (SCD) in older adults is one of mechanism rather than of phenomenology [61]. SCD with biomarker positivity for Alzheimer’s disease carries genuine risk of progression; SCD with intact metacognitive sensitivity and normal biomarkers more closely resembles FCD [61]. This positions metacognitive efficiency as a potential diagnostic and prognostic marker—a point we return to in Section 7.

## 4. Persistent Post-Concussion Symptoms (PPCS) as a Disorder of Distributed Memory Control

Post-concussion symptoms, particularly in their persistent form (PPCS), are conventionally defined by a cluster of physical, cognitive and emotional symptoms continuing beyond the expected recovery window after mTBI [4,6,8,12,13]. Memory and concentration complaints are nearly universal in this group [6,8,13]. Yet, as in FCD, formal neuropsychological testing in PPCS often reveals only modest deficits, particularly on speeded and attentional measures rather than on classical declarative memory tasks [6,8,12,62]. The framework developed in this paper requires us to take the initial biological injury seriously while also explaining why, in a subset of patients, symptoms persist long after the underlying perturbation should have resolved.

### 4.1. The Initial Brain Injury: Neurometabolic Cascade and Distributed Network Insult

It is essential to be unambiguous on this point: the brain injury in mTBI is real, biological and measurable, even when conventional structural imaging is normal. Concussive biomechanical forces produce mechanical stretching of axons and disruption of neuronal membranes, opening voltage-gated potassium channels and producing a marked efflux of intracellular potassium [45,63,64]. This nonspecific depolarisation triggers an indiscriminate release of excitatory amino acids—most prominently glutamate—which further activates NMDA, AMPA and kainate receptors, drives calcium influx, and propagates the disturbance across neuronal populations [45,63,64]. The Na^+^/K^+^-ATPase responds with a surge in energy demand precisely as mitochondrial function is impaired and cerebral blood flow is reduced, generating the well-described acute energy crisis of concussion [63,64]. An inhibitory “spreading depression -like phase follows, with global metabolic depression that can persist for days [63,64].

***Evidence from animal studies:*** Crucially for the present argument, the neurometabolic cascade directly perturbs the plasticity machinery on which the distributed memory control system depends. NMDA receptor subunit composition is altered after TBI, with corresponding changes in receptor function. These changes have downstream effects on CaMKII, ERK and CREB signalling and on BDNF-dependent structural plasticity [45,46,63]. The molecular processes involved are the same ones that underlie long-term potentiation, systems consolidation, and the maturation of cortical engrams [17,18,27,45]. However, this evidence comes entirely from animal studies; the relevant molecular changes have not been demonstrated in human mTBI, and the idea that these processes translate into memory disorders after a concussion is based on inference rather than direct observation. In the acute and subacute window, therefore, mTBI does not simply produce diffuse symptoms; it specifically disrupts the cellular substrate of memory stabilisation.

***Evidence from human studies:*** In humans, the cascade is inferred rather than being measured directly; support for this comes from magnetic resonance spectroscopy, which shows altered ratios of N-acetylaspartate and metabolites in the days following a concussion, from evidence of reduced cerebral blood flow, and from the clinical course of the acute symptoms, which conforms to the pattern that the animal studies predict [63,64]. However, no human study has shown the receptor-level or kinase-level changes mentioned above.

***Hypotheses and predictions:*** The idea that this cascade specifically interferes with the cellular substrate of memory stabilisation, rather than causing widespread dysfunction, is based on the convergence of the molecular pathways concerned. It suggests that memory-specific deficits during the subacute phase should be more closely related to markers of metabolic recovery than to indices of injury severity.

The duration of this window can be specified, and its overlap with the psychological window is central to the hypothesis. In rodent models, the metabolic depression and impaired plasticity of the cascade persist on the order of seven to ten days [63,64]; NMDA-receptor subunit protein is acutely reduced within hours before recovering, whereas the more prolonged and plasticity-relevant change is a shift in subunit composition toward immature assemblies that impair long-term potentiation during recovery [64,65]. In humans, magnetic-resonance spectroscopy shows neuronal-metabolic integrity still depressed at fifteen days and normalising only around thirty days after a single concussion—importantly, this metabolic recovery outlasts symptom resolution and is prolonged further by re-injury within the window [66]. The significance for the present framework is the temporal coincidence: this biological window of impaired plasticity—days to several weeks—overlaps almost exactly with the psychological window of acute illness anxiety, hypervigilant symptom monitoring and disturbed sleep. This seems to present a certain difficulty since, if plasticity is impaired, the ability to form any durable model—even a maladaptive one—should be diminished rather than increased. We suggest that the impairment is selective not global. Of the various processes affected during the acute phase, it is those which support accurate, iterative corrective updating that are most impacted: sleep-dependent consolidation, dream-mediated regularisation and the metabolic reserve needed for the effortful re-encoding of performance data relevant to the self. The encoding of a single, highly salient, affectively charged and repeatedly rehearsed illness narrative relies less on these mechanisms. Our proposal is therefore not that overall plasticity rises, but that the balance between corrective updating and salience-driven consolidation shifts in favour of the latter, so that whatever is written during this period is much more likely to be the illness model than an accurate one. We put forward this as a hypothesis; it has not yet been directly tested, and it would be refuted if it were found that both corrective updating and salience-driven consolidation are similarly impaired in the post-concussive period.

At the network level, modern neuroimaging has moved decisively away from focal-lesion models of mTBI. Resting-state functional MRI studies repeatedly identify altered connectivity within the default mode network, the salience network and thalamo-cortical circuits, with reduced functional connectivity persisting into the chronic phase in patients with PPCS [15,62,67]. Diffusion imaging shows widespread microstructural changes that may be invisible on conventional MRI [68]. Sleep architecture is reliably disturbed in a high proportion of mTBI patients, with implications both for consolidation and for the regularising function of dreaming discussed in Section 3.5 [69,70]. Attention, processing speed and working memory—the capacities that support prefrontal retrieval and metacognitive monitoring—are reliably affected in the early period after injury [8,12,62].

Two implications follow. First, the acute mTBI insult is precisely the kind of perturbation predicted to disrupt distributed memory control: thalamo-cortical rhythms, sleep-dependent consolidation, attentional resources and the plasticity machinery of the encoder–stabiliser system are all affected simultaneously. Second, the injury creates a window of network vulnerability during which the metacognitive controller—itself dependent on these processes—is uniquely susceptible to miscalibration.

### 4.2. Reasons for PPCS to Persis

The clinical puzzle of post-concussion symptoms is not the acute presentation but their persistence. The majority of patients recover within weeks to months; a substantial minority do not [6,8,12]. We have previously argued, in the Functional Overlay Model of PPCS, that persistence reflects a layered process: an initial neurobiological injury sets up a vulnerable system, and subsequent psychological, behavioural and physiological factors maintain symptoms long after the primary injury would otherwise have resolved [13,14,15]. Picon, Silverberg and colleagues have provided empirical support, showing that memory perfectionism, depression and prior psychiatric history predict persistent memory complaints after concussion more strongly than initial injury characteristics or objective neuropsychological performance [71,72]. The size of this group is now well documented. In the TRACK-TBI cohort, only 27% of patients with mTBI had fully recovered function at two weeks and 53% still reported injury-related functional limitation at twelve months, despite minimal structural injury [73]; a systematic review and meta-analysis estimates persistent post-concussion symptoms in roughly one in six patients at three to six months, with unadjusted estimates approaching one in three [74]. Psychiatric overlay is comparably common, with probable post-traumatic stress disorder or major depression in about one in five mTBI patients at three to six months—roughly double the rate in orthopaedic-injury controls [75]. Persistent disability is better predicted by psychological and expectational factors than by the severity of the injury [71,72]. This association is consistent with a biological gateway-to-functional-disorder model, but it is predictive rather than causally proven. There are two exceptions, however. The severity measures for mTBI are crude and may not reflect clinically significant differences in the extent of injury, so the comparison is not between two variables measured with equal accuracy. Also, the psychological factors in question—anxiety, illness beliefs, and perfectionism—might have existed before the injury rather than having developed as a result of it; in that case, they indicate a predisposition rather than representing the actual cause. To determine which of these two possibilities is correct, prospective studies with measurements taken before the injury would be needed.

Within the distributed memory control framework, several converging mechanisms maintain PPCS-related memory symptoms. Sleep disturbance—including insomnia, fragmented sleep and altered slow-wave activity—directly impairs systems consolidation and thalamo-cortical dialogue and degrades the dream-mediated regularisation discussed in Section 3.5 [25,26,43,44,69,70]. Fatigue and impaired processing speed limit the prefrontal resources available for cue-guided retrieval, so that traces that exist in cortex cannot be accessed efficiently [57,62,76]. Attentional hypermonitoring of cognitive symptoms—encouraged by clinical messaging that frames concussion as fragile and high-stakes—recruits the same self-referential and monitoring networks that, in FCD, become aberrantly engaged [9,14,15,57]. Memory perfectionism, identified empirically as a predictor of persistent symptoms after concussion [71,72], drives the same overfitted self-model described in Section 3.5: the patient measures every cognitive event against an idealised standard, treats normal lapses as evidence of injury, and shrinks their experiential distribution accordingly.

### 4.3. Concussion as a Gateway to Functional Cognitive Disorder

We have recently argued, on the basis of converging clinical and neuropsychological evidence, that mTBI may act as a gateway condition for the development of FCD proper in vulnerable individuals [30]. The clinical motivation is straightforward: a subset of patients presenting after concussion go on to develop persistent and disproportionate autobiographical memory disturbance that exceeds expected neuroanatomical limits, lacks structural correlates on neuroimaging, and in rare cases resembles extensive retrograde or identity-related amnesia [30]. These presentations are not adequately explained by neurometabolic injury—which is reversible and limited in extent—nor by malingering, which the literature does not support as a primary mechanism in this population [9,11,57,72,77].

The gateway hypothesis articulates a mechanism. Pre-existing predisposing factors—perfectionism, anxiety, prior psychological trauma, somatisation tendencies, illness anxiety, prior FND or chronic pain—exist in many individuals at subclinical levels and would not, on their own, produce a functional cognitive syndrome. The mTBI provides a precipitant of a very specific kind: it transiently disrupts the very network that supports metacognitive calibration, attention and sleep-dependent regularisation, while simultaneously providing a salient explanatory narrative (“I have brain damage”) that the metacognitive controller can latch onto. In the recovery window, the system is asked to relearn what its own cognitive performance looks like—and it does so against a background of fatigue, sleep disruption, illness anxiety and pre-existing perfectionism. In vulnerable individuals, the network does not relearn an accurate self-model; it overfits, in the sense of Section 3.5, to a narrow, illness-centred distribution. The functional cognitive syndrome that results is then maintained by the same metacognitive, attentional and behavioural mechanisms that maintain primary FCD [3,15,30,59]. It is important to recognise a considerable gap in this area: there has been no direct study of metacognitive function during the acute and subacute phases following a mTBI. The idea that metacognitive calibration is disturbed during this period is based on the existing body of research concerning network disruption and has not been directly measured. A direct evaluation of metacognitive accuracy—for instance, assessing trial-by-trial confidence calibration on memory tasks in the first weeks after injury—should be a key, readily attainable focus of future research and would constitute one of the most direct ways to test the gateway hypothesis.

This account predicts a continuum between primary FCD, mixed PPCS/FCD presentations and (in rare cases) florid functional retrograde or dissociative amnesia [30]. It also predicts that the risk of progressing along this continuum is highest in patients with multiple predisposing factors, with prolonged early sleep disruption and fatigue, and with clinical environments that inadvertently reinforce illness narratives.

These predictions can be tested in prospective cohorts recruited immediately after a concussion, a type of cohort already established in sports and military contexts. Because the predisposing factors at the heart of the gateway hypothesis are, by definition, pre-injury, they can be assessed prospectively only if a high-risk group is enrolled before injury. A structured history obtained from family members or close associates therefore provides a practical solution, enabling retrospective assessment of any previous perfectionism, illness anxiety, and functional symptoms, as well as independent verification of the circumstances surrounding the injury and the time relationship between the injury and the onset of symptoms. Collateral interviewing of this kind is already a standard procedure in the management of functional disorders and we suggest that it should be included as part of any evaluation of this spectrum.

Even though the continuum has been put forward rather than proven, it is still possible to lay down provisional criteria for placing patients along it, at least to make the proposal testable. Three dimensions seem to be the most helpful. The first of these is whether there has been a documented injury—that is, a mTBI which has either been witnessed or recorded by medical professionals—rather than one that has simply been reported. The second dimension concerns the temporal relationship between the injury and the onset of symptoms—namely, whether the cognitive complaints began during the acute period and persisted, or whether they appeared after a period of recovery. The third is the pattern of internal inconsistency observed during examination: the variation between performance when spontaneous and when formally assessed, and the dissociation between local and global metacognitive accuracy, a difference which we would anticipate to become greater as one moves towards the FCD end of the continuum. According to this approach, primary FCD is marked by either the absence or the remoteness of the injury, by a delayed or unrelated onset of symptoms, and by a high degree of internal inconsistency; mixed cases are those in which there is a documented injury, symptoms persist beyond the expected period of recovery, and inconsistency also begins to appear; and predominantly organic PPCS involves a documented injury, continuous symptoms from the outset, and only minimal inconsistency. We must emphasise that these are heuristics derived from the framework, not validated diagnostic rules, and that their sensitivity and specificity are unknown. They are being presented as a testable suggestion for future evaluation, not as a clinical tool.

### 4.4. Recognising the Overlay Without Dismissing the Injury

It is essential that the overlay and gateway models are not heard as a return to the idea that PPCS is “psychological”. The neurobiological injury is real, measurable in connectivity, microstructure, neurochemistry and plasticity machinery [13,14,15,62,63,64,67,68], and clinically meaningful. What the model claims is that the persistence of symptoms in a particular patient is rarely explicable by the residual injury alone, and that fatigue-related, sleep-related, metacognitive and behavioural processes—each tied to identifiable nodes of the memory control system and to identifiable cellular substrates—are essential to a full account [13,14,15,30].

## 5. Neuroplasticity as the Substrate of Dysfunction and Recovery

The framework developed so far identifies disturbed control of a distributed memory network as the proximate cause of memory symptoms in FCD and PPCS. We now argue that the more distal cause—the reason these disturbances persist rather than self-correct—is maladaptive neuroplasticity.

Neuroplasticity is not a single phenomenon but a family of processes operating across scales: NMDA-dependent long-term potentiation and depression at individual synapses; structural remodelling of dendritic spines mediated by BDNF and downstream kinase cascades; sleep-dependent synaptic homeostasis that renormalises plasticity nightly; and large-scale functional reorganisation across cortical networks [27,28,45,46]. These processes are normally adaptive; they are the cellular currency of learning, memory consolidation and recovery from injury. But the same machinery that allows the brain to learn can entrench dysfunctional patterns—a principle increasingly recognised across the FND spectrum [27,28].

### 5.1. Maladaptive Plasticity in FCD and FND

***Evidence from human studies:*** In a recent synthesis of FND as a disorder of maladaptive plasticity, the picture is one of dynamic brain states shaped by aberrant neuroplastic mechanisms rather than fixed structural lesions [27,28]. Functional symptoms can be conceptualised as reinforced symptom circuits—patterns of activity that have been strengthened by repetition, attention, expectation and emotional salience, and that come to act as learned templates [11,27,28]. In FCD specifically, the repeated retrieval of negative self-evaluations (“I forgot another name today”), pairing of normal mnemonic noise with anxiety, and rehearsal of illness narratives in clinical encounters and online forums all provide repeated activation of the same metacognitive prediction–evaluation circuits. Each rehearsal strengthens the synaptic substrate of those circuits; the overfitted internal model of Section 3.5 is, at the cellular level, a use-dependent model.

This micro-synaptic account should, if correct, leave a macroscopic signature in functional connectivity—and in functional neurological disorder, it does. Among more consistently reported findings is heightened coupling between limbic-affective and motor-preparatory cortex: increased amygdala–supplementary-motor-area connectivity during emotional arousal, amygdala directly influencing motor cortex [78]; the coupling is symptom-specific and state-dependent [79] and its strength scales with symptom severity in the corticolimbic ‘fast-tracking’ model [80]. A complementary abnormality reduces temporoparietal-junction connectivity to sensorimotor cortex, degrading the feed-forward/feedback comparison thought to underlie the sense of agency [81]. Notably, the changes take place despite the overall macroscopic anatomy remaining largely unaltered. The distinction we are making is not one between function and structure—for function is in the end determined by structure at some level of description—but between different levels of structural organisation: namely, changes in synaptic efficacy, receptor composition and postsynaptic organisation, all of which are structural, and the preservation of the general tissue integrity. This is what one would expect to see in the case of use-dependent reinforcement rather than in the case of tissue loss [77].

***Hypotheses and predictions:*** Dedicated resting-state connectivity studies in functional cognitive disorder are still lacking; we therefore advance these emotion–motor and agency-network findings as the empirical template and treat their extension to the retrieval circuitry of FCD as a testable prediction rather than an established result.

This view connects naturally to the predictive processing account [9,24,56]. Aberrantly precise priors are not simply abstract beliefs; they are implemented in synaptic weights. The failure to update priors, on this reading, is a failure of plasticity to move the relevant weights—either because contradictory evidence is filtered out before it reaches the relevant synapses, or because the recurrent activation of the existing pattern outcompetes weaker novel signals.

### 5.2. Plasticity After mTBI

The cellular evidence here is that of Section 4.1 and is animal-derived; what follows is its extension to human recovery, which is hypothetical. The plasticity story is also central to PPCS.

***Evidence from animal studies:*** As outlined in Section 4.1, the neurometabolic cascade after concussion alters NMDA receptor function and downstream kinase signalling and disturbs BDNF-mediated structural plasticity [45,46,63]. The acute consequence is a transient impairment of the very machinery that mediates new learning, consolidation and adaptive reorganisation.

***Hypotheses and predictions:*** In most patients, this resolves; in a vulnerable subgroup, recovery occurs against a background of disturbed sleep, fatigue and illness anxiety. The plasticity machinery is, in effect, brought back online in an unhelpful learning environment, and what it learns is a maladaptive symptom pattern [13,14,27]. Sleep disturbance is particularly important here, because sleep-dependent synaptic homeostasis is the normal mechanism by which the brain renormalises plasticity and prevents over-fitting to recent experience [25,43]; when it is impaired, dysfunctional learning is preferentially preserved.

### 5.3. Plasticity as a Route to Recovery

The same plasticity that entrenches dysfunction is the substrate of recovery. This is the rationale, often implicit, for cognitive rehabilitation, metacognitive therapy, physiotherapy in motor FND and psychoeducation: each provides repeated, structured experiences capable of reshaping synaptic weights toward more adaptive patterns [27,28]. Behavioural experiments that violate predicted-failure priors generate prediction errors that, in a system with intact plasticity machinery, can update those priors. Graded successful retrieval—for example, learning that one can in fact remember a list—provides use-dependent reinforcement of accurate self-models. Sleep optimisation restores the regularising and renormalising functions of slow-wave and REM sleep [25,43,44]. The shift from “these symptoms are functional, therefore not real” to “these symptoms are maintained by maladaptive plasticity in identified circuits, and plasticity is the same mechanism by which we will treat them” is, in our view, the central conceptual move that the field now needs to make.

## 6. A Unified Metacognitive–Systems Consolidation Framework

The Functional Overlay Model and the suggestion that a concussion might serve as a gateway to a functional cognitive disorder are based on our earlier theoretical research [13,14,30] and are included in this paper as background information and not as independent empirical evidence. The present manuscript is not merely a restatement of those earlier proposals; rather, it makes three contributions: it combines the distributed memory-control architecture—comprising anteromedial thalamic gating, prefrontal retrieval and metacognitive evaluation, and sleep-dependent consolidation—with the overfitting explanation of self-model formation, which was not considered in the previous work; it specifies the cellular and temporal mechanism through which the two pathways come together, identifying the point of convergence as being in the overlap between the post-concussive plasticity period and the period during which a durable self-model is formed; and it draws from this integration a number of specific, falsifiable predictions (in Section 7) and provisional criteria for placing patients on the proposed continuum (in Section 4.3), neither of which can be derived solely from the earlier models.

The argument so far is that both FCD and PPCS converge on dysfunction within the same distributed memory control system, but affect the system through different mechanisms.

In FCD, the dominant disturbance is top-down: aberrantly precise prior beliefs about cognitive failure, distorted global metacognition, excessive monitoring of normal mnemonic noise, and overfitting of the internal self-model under conditions of impaired sleep and dream-mediated regularisation [1,3,9,11,25,50,51,57,59]. The cortical store and the hippocampal–thalamic encoder–stabiliser system are essentially intact; the metacognitive controller is miscalibrated, and the controller’s miscalibration is use-dependently maintained.

In PPCS, the entry point is bottom-up: a real neurometabolic and network injury disturbs attention, processing speed, thalamo-cortical rhythms, sleep-dependent consolidation and the plasticity machinery itself [12,15,45,62,63,64,69,70]. The encoder–stabiliser system is genuinely perturbed in the acute phase. In the subset of patients who progress to persistent symptoms, fatigue, hypermonitoring, memory perfectionism and an emerging functional overlay maintain the disturbance and eventually engage the same metacognitive miscalibration and overfitted self-model that characterise FCD [13,14,30,71,72]. In particularly vulnerable individuals, the concussion functions as a gateway, organising pre-existing predisposing factors into a stable functional cognitive syndrome [30].

Convergence on a common phenomenology is the observation that the framework is constructed to explain, rather than a prediction derived from it: FCD and PPCS arise by different routes yet present similarly. Three implications arise from this approach. First, the framework predicts a continuum rather than a dichotomy between FCD and PPCS, with functional cognitive symptoms associated with mTBI occupying intermediate positions [14,30,71,72]. Second, maladaptive plasticity is the common cellular substrate that links the two routes: top-down miscalibration in FCD and bottom-up injury in PPCS both ultimately entrench dysfunctional patterns through the same plasticity machinery the system uses for learning [27,28]. Third, a useful clinical taxonomy must distinguish patients dominated by genuine early network disruption from those dominated by metacognitive miscalibration and overfitted self-models, because the targets—and timing—of treatment differ.

**Figure 2:**
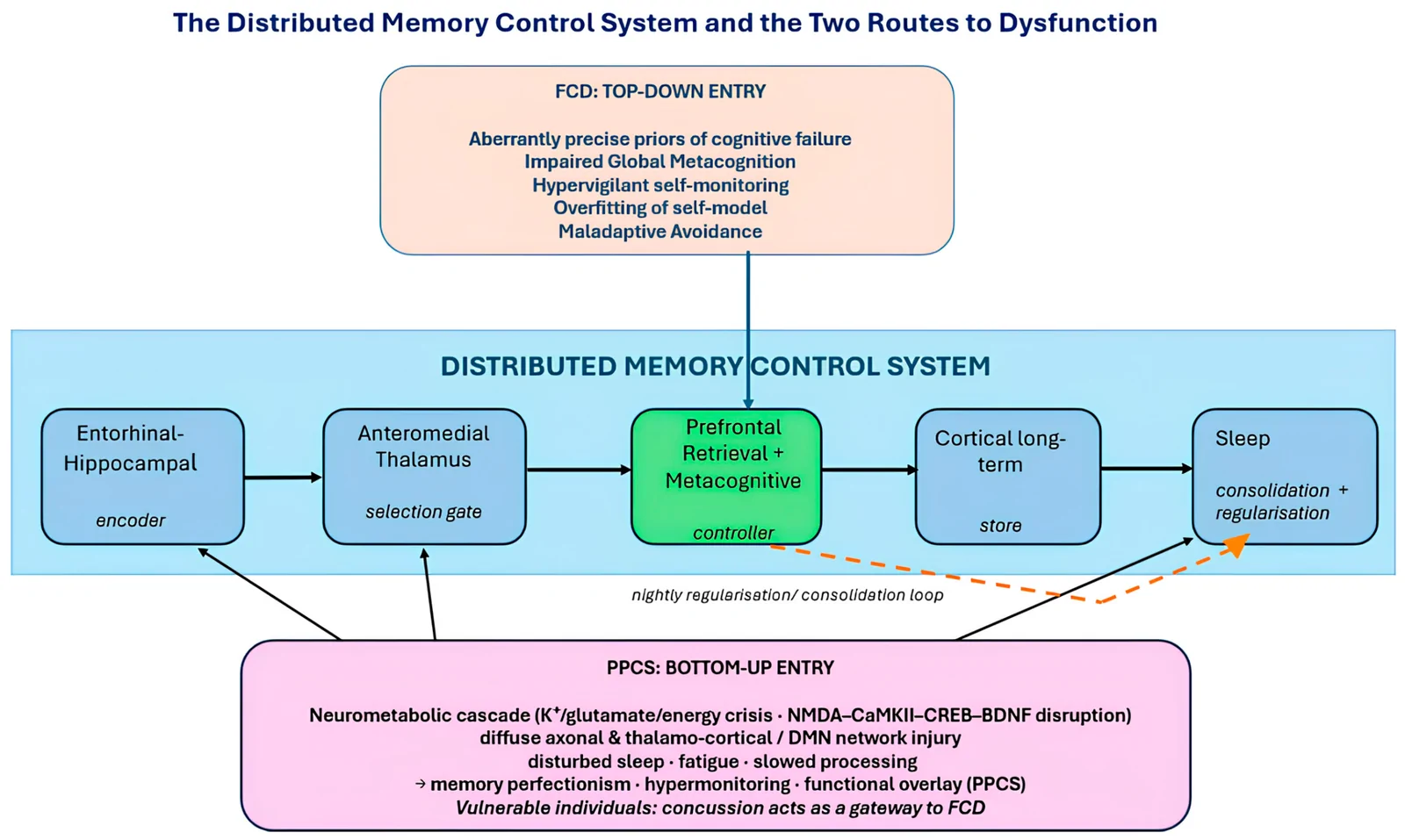
Proposed architecture of the distributed memory control system and the two routes to dysfunction. Entorhinal–hippocampal encoder → anteromedial thalamic selection gate → prefrontal retrieval and metacognitive controller → cortical long-term store, with a sleep-dependent consolidation and regularisation loop. Functional cognitive disorder is proposed to enter top-down through miscalibration of the metacognitive controller; persistent post-concussion symptoms (PPCS) are proposed to enter bottom-up through neurometabolic and network injury. Maladaptive, use-dependent plasticity is proposed as the shared substrate entrenching both routes. The thalamic gating function shown is established for consolidation in animal studies and hypothesised for retrieval in humans (Section 2.2).

A schematic of this framework is presented in Figure 2, and Table 1 summarises the contrast between the two routes across the dimensions of the model. It depicts the hippocampal–entorhinal encoder, the anteromedial thalamic gate, the prefrontal retrieval and metacognitive controller, the cortical long-term store, and the sleep-dependent consolidation/regularisation loop. FCD enters from a top-down miscalibration of the metacognitive controller (aberrant priors, overfitting, hypermonitoring); PPCS enters from a bottom-up perturbation of attention, sleep, thalamo-cortical rhythms and plasticity machinery; both routes converge on the same maintenance loop through maladaptive plasticity.

## 7. Testable Predictions

The framework yields a set of empirically testable predictions, several of which distinguish it from alternative accounts.

(1) Recognition–recall dissociation. Both groups should show preserved or near-preserved recognition memory but disproportionately impaired free recall, reflecting prefrontal retrieval inefficiency rather than hippocampal storage failure [19,57]. The pattern should be more pronounced in FCD than in the early post-concussion period, and should emerge in late PPCS as functional overlay develops. This is decisive in a single case–control study: equivalent impairment of recognition and free recall, or a recall deficit that correlates with an encoding rather than a retrieval-monitoring index, would falsify the storage-intact claim.

(2) Local–global metacognitive dissociation. FCD patients should show intact or near-intact local (trial-by-trial) metacognitive efficiency but markedly impaired global self-evaluation [1,42]. Patients with PPCS should show an intermediate pattern, with global metacognitive distortion emerging over time. The prediction is falsifiable in one metacognition study: if global self-evaluation is intact or is impaired to the same degree as local metacognitive efficiency, the dissociation is refuted.

(3) Confidence–performance miscalibration. Both groups should show a flattened or inverted relationship between objective task performance and subjective confidence, with effect sizes large enough to distinguish them from healthy controls and from neurodegenerative MCI [40,41,61]. A single calibration study would refute this if FCD and PPCS confidence–performance slopes were statistically indistinguishable from those of healthy controls.

(4) Overfitting signatures. If FCD reflects overfitting of internal models, then FCD patients should show (a) impaired generalisation on transfer tasks despite preserved memorisation of trained stimuli; (b) reduced REM/dreaming indices on polysomnography or dream-report measures; and (c) disproportionate reduction in memory complaints following interventions that broaden the distribution of cognitive evidence (behavioural experiments, graded retrieval success) compared with interventions targeting depression or anxiety alone [25,26,59]. The prediction is refuted if FCD patients generalise normally on transfer tasks despite an active memory complaint, or if REM/dreaming indices are normal on polysomnography.

(5) Fatigue- and sleep-dependent variability. PPCS-related memory performance should be more sensitive than FCD-related memory performance to manipulations of fatigue, time of day and sleep quality, reflecting the underlying network vulnerability [25,43,44,57,62,69,70]. A single within-subject manipulation of sleep or fatigue would falsify this if PPCS-related and FCD-related performance proved equally (in)sensitive to it.

(6) Effort and processing speed. Subjective mental effort should exceed objective task demand in both groups, with the dissociation most pronounced in FCD and predicting global metacognitive distortion [50,57]. It is refuted if subjective effort matches objective task demand, or if the effort–demand gap fails to predict global metacognitive distortion.

(7) Network-level imaging signatures. We predict reduced functional connectivity within thalamo-cortical and default mode networks in PPCS [15,62], with relative preservation of these networks in FCD; conversely, altered activity in anterior prefrontal and precuneal regions implicated in metacognition [37,38,82] should be more prominent in FCD. A single resting-state fMRI study would falsify this if thalamo-cortical and default-mode connectivity were reduced equally in FCD and PPCS, or if metacognition-related prefrontal/precuneal activity did not differ between them.

(8) Plasticity-linked treatment response. FCD-pattern presentations should respond preferentially to metacognitive therapy, sleep optimisation and behavioural experiments that target the overfitted self-model [27,28,57,58]; PPCS-pattern presentations should respond preferentially to early relative rest with graded return to activity, sleep treatment, pacing and graded re-exposure [13,14,69,73]; mixed presentations should require combined strategies. Recovery in both groups should be associated with measurable changes in connectivity and, where assessable, in markers of synaptic plasticity. It is refuted if the two presentations respond equally to the mechanistically opposite treatments, or if clinical recovery occurs with no measurable change in connectivity or plasticity markers.

Table 2 summarises these predictions together with the comparisons that would discriminate the framework from alternative accounts.

## 8. Differential Diagnosis

Early neurodegeneration, particularly amnestic mild cognitive impairment and prodromal Alzheimer’s disease, can present with memory complaints and is the most consequential differential. The distinguishing features are objective rather than subjective: progressive impairment on delayed recall and recognition, biomarker positivity (CSF or imaging), and structural change on MRI [2,6,61]. Metacognitive sensitivity tends to be relatively preserved early in neurodegeneration, then progressively impaired as anosognosia develops—the opposite trajectory to FCD, where global metacognition is impaired but local sensitivity preserved [1,42,61].

Depression and anxiety frequently produce subjective cognitive complaints and may be the primary driver in some patients. They are not, however, mutually exclusive with FCD or PPCS; comorbidity is the rule [2,3,8,13,72]. A pragmatic approach is to treat affective comorbidity systematically and reassess cognitive complaints once they have remitted. Persistence of complaints with intact objective performance after effective mood treatment is suggestive of an additional FCD component.

ADHD, obstructive sleep apnoea and other sleep disorders, thyroid dysfunction, medication effects and substance use should be considered and excluded in standard fashion. None of these is incompatible with the framework, but each can drive symptoms independently and may attenuate or mimic the predicted profile.

The contentious issue of effort and performance validity testing deserves explicit acknowledgement. Teodoro et al. found that performance validity test failure occurred in only a minority of patients across FND, fibromyalgia and chronic fatigue syndrome [57], and the conflation of functional cognitive symptoms with feigning is conceptually and empirically untenable [9,11,24,72]. Performance validity testing is a useful adjunct, not a substitute for diagnosis, and should be interpreted within the broader clinical pattern.

## 9. Clinical Implications

The following recommendations fall into two categories, and we make this distinction clear. Section 9.1 describes the approaches for which there is already evidence in FCD, functional neurological disorder or PPCS, specifying the extent of that evidence in each instance. Section 9.2 presents interventions suggested by the current framework but not yet tested in these groups; these are hypotheses regarding treatment rather than clinical recommendations and are accompanied by measurable objectives by which they could be assessed. It is not claimed that the framework has established clinical utility, as such a conclusion would require a prospective evaluation.

### 9.1. Approaches with Existing Evidence

The sleep treatment aims at both the consolidation and the regularisation nodes; cognitive-behavioural therapy for insomnia, the assessment of sleep-disordered breathing and the management of post-traumatic sleep fragmentation are included within the clinically indicated management for this group [25,43,44,69,70]. According to our framework, the benefits from sleep treatment will be notably greater in PPCS as compared with those from interventions directed at the other nodes; however, such a comparison has not yet been carried out, and the prediction is given here. In the management of persistent symptoms following a mild traumatic brain injury, pacing and a graded return to activity are clinically recommended; the suggestion that they work by breaking the cycle in which self-monitoring deteriorates the performance it is intended to measure is a mechanistic explanation provided by this framework, not a proven mode of action.

It should be made clear that there is no pharmacological neuroprotective agent which has been shown to provide any benefit in the case of mild traumatic brain injury; current management during the acute phase is behavioural and involves a short period of relative rest followed by a stepwise, symptom-limited recovery back to activity [73].

Trauma-informed care is called for since a large number of patients who have persistent symptoms have experienced psychological trauma, have gone through adverse life events or have had a previous psychiatric illness whereby these factors interact with the injury [13,14,30,72]. Explanatory clinical communication is part of the management of functional disorders when clinical indications are present, and its value has been shown in the case of functional neurological disorder more generally [11,51,58]. The idea of viewing FCD and PPCS as disorders of memory control, maintained by plasticity processes that the same brain can also employ for recovery, is in line with this approach. However, it has not been tested whether this specific way of framing leads to better outcomes than the current explanatory models.

### 9.2. Framework-Derived Hypotheses

Mapping the clinical implications onto the framework yields specific, mechanism-linked interventions. Metacognitive therapy and psychoeducation target the metacognitive controller and the overfitted self-model. They address aberrantly precise priors about cognitive failure, memory perfectionism and catastrophic interpretation of normal mnemonic noise, and they provide the broadened experiential distribution that an overfitted system requires for recalibration [27,51,57,58,72]. Cognitive rehabilitation targets prefrontal retrieval and attentional control; strategies that train attentional flexibility, reduce dual-task self-monitoring and rebuild confidence through graded successful encoding and retrieval are predicted to be most useful in late PPCS and in mixed presentations [12,13,14,15].

Table 3 maps these interventions onto the nodes of the memory control system and indicates the current evidence status of each.

## 10. Conclusions

We have proposed that the mismatch between severe subjective memory complaints and near-normal objective testing in FCD and PPCS may be better understood not as a failure of hippocampal storage but as a disturbance of a distributed memory control system, maintained by maladaptive neuroplasticity. Anteromedial thalamic gating, prefrontal retrieval and metacognitive evaluation, cortical long-term storage, and sleep-dependent consolidation and regularisation together constitute the architecture whose disruption—entered top-down in FCD, bottom-up in PPCS, and via concussion as a gateway in vulnerable individuals—generates the observed phenomenology. Reading FCD as an overfitting disorder integrates the metacognitive, prior-update, attentional and behavioural mechanisms that have been described in the literature; recognising the neurometabolic and plasticity-level substrate of mTBI clarifies why the brain is uniquely vulnerable to overlay in the post-injury window. This metacognitive–systems consolidation framework, scaffolded by maladaptive neuroplasticity, integrates recent circuit-level neuroscience with the functional disorder literature, generates testable predictions, and points toward mechanism-specific interventions.

The framework put forward in this paper is suggested as a hypothesis to be tested and not as a final explanation. Its merit is due to the specificity of the predictions it makes and to its falsifiability, and it should be assessed according to whether these predictions withstand future evaluation.

## Figures and Tables

**Table 1:** Proposed distinctions between functional cognitive disorder and persistent post-concussion symptoms across the dimensions of the framework. Entries describe hypothesised rather than empirically established contrasts, except where supported in the text.

Dimension	Functional Cognitive Disorder	Persistent Post-Concussion Symptoms
Entry point	Top-down (metacognitive controller)	Bottom-up (neurometabolic + network injury)
Initial substrate	Aberrantly precise priors; overfitted self-model	K^+^/glutamate cascade; NMDA–CaMKII–CREB–BDNF disruption; diffuse axonal injury
Encoder/store	Essentially intact	Genuinely perturbed in acute phase
Memory profile	Recall ≪ recognition; internal inconsistency	Early: speed/attention. Late: recall ≪ recognition as overlay develops
Metacognition	Impaired global, preserved local	Intermediate; global distortion emerges over time
Sleep sensitivity	Lost dream-mediated regularisation	Marked: fragmented sleep degrades consolidation + regularisation
Maintaining loop	Hypermonitoring, perfectionism, avoidance	Fatigue, hypermonitoring, perfectionism, functional overlay
Shared substrate	Maladaptive (use-dependent) plasticity	Maladaptive (use-dependent) plasticity
Primary treatment lever	Metacognitive therapy; broaden evidence distribution; sleep	Early relative rest with graded return to activity; sleep treatment; pacing; graded re-exposure

**Table 2:** Testable predictions and the methods that would discriminate the framework from alternative accounts. ↓ denotes a predicted reduction. aPFC, anterior prefrontal cortex; DMN, default mode network; rs-fMRI, resting-state functional magnetic resonance imaging.

#	Prediction	Method/Discriminating Comparison
1	Recognition–recall dissociation	Recognition vs. free recall; FCD > early post-concussion period; emerges in late PPCS
2	Local–global metacognitive dissociation	Hierarchical Bayesian meta-d′ vs. MMQ; FCD intact local/impaired global
3	Confidence–performance miscalibration	Type-2 ROC vs. HC and amnestic MCI; opposite trajectory to MCI
4	Overfitting signatures	Transfer/generalisation tasks; REM & dream indices; response to distribution-broadening interventions
5	Fatigue/sleep-dependent variability	Performance × time-of-day × sleep manipulation; PPCS > FCD sensitivity
6	Effort exceeds objective demand	Subjective effort vs. RT/load; largest in FCD; predicts global distortion
7	Network imaging signatures	rs-fMRI: thalamo-cortical/DMN ↓ in PPCS; aPFC/precuneus altered in FCD
8	Plasticity-linked treatment response	Stratified trials; recovery tracks connectivity/plasticity markers

**Table 3:** Mechanism-linked interventions mapped to nodes of the memory control system, with the current evidence status of each. Entries in the Targeted node column state the mechanism proposed by this framework; they are not established modes of action.

Intervention	Targeted Node/Mechanism	Evidence Status
Metacognitive therapy & psychoeducation	Metacognitive controller; aberrant priors; overfitted self-model	Evidence in FND and health anxiety; not tested in FCD
Behavioural experiments/graded retrieval success	Generates disconfirming prediction error; broadens evidence distribution	Hypothesised
Cognitive rehabilitation	Prefrontal retrieval & attentional control; reduces dual-task self-monitoring	Evidence in PPCS; proposed mechanism hypothesised
Sleep treatment (CBT-I, OSA management)	Consolidation + dream-mediated regularisation nodes	Clinically indicated; regularisation mechanism hypothesised
Pacing & graded exposure	Fatigue-monitoring loop	Clinically indicated; proposed mechanism hypothesised
Early relative rest and graded return to activity (PPCS)	Neurometabolic cascade window; plasticity machinery	Consensus-based [73]
Trauma-informed care	Predisposing factors; gateway risk in vulnerable individuals	Evidence-informed
Explanatory clinical communication	Reframes symptoms as plasticity-maintained and treatable	Evidence in FND; untested in FCD

## Data Availability

Data sharing is not applicable to this article, as no new data were created or analysed. All sources discussed are available in the cited published literature.

## Abbreviations

BDNF, brain-derived neurotrophic factor; CaMKII, calcium/calmodulin-dependent protein kinase II; CBT-I, cognitive behavioural therapy for insomnia; CREB, cAMP response element-binding protein; DMN, default mode network; FCD, functional cognitive disorder; FND, functional neurological disorder; MCI, mild cognitive impairment; MMQ, Multifactorial Memory Questionnaire; mTBI, mild traumatic brain injury; NMDA, N-methyl-D-aspartate; OSA, obstructive sleep apnoea; PPCS, persistent post-concussion symptoms; REM, rapid eye movement; rs-fMRI, resting-state functional magnetic resonance imaging; SCD, subjective cognitive decline.
